# Supplementary material for: Impacts of Climate Change on the Habitat Suitability and Natural Product Accumulation of the Medicinal Plant Sophora alopecuroides L. Based on the MaxEnt Model
Source: Plants (Basel). 2024 May 21;13(11):1424. doi: 10.3390/plants13111424 (PMC11174999; doi:10.3390/plants13111424)
Supplement: Supplementary file 1 [file plants-13-01424-s001.zip › plants-2916375-supplementary.pdf]

Article

Impacts of climate change on habitat suitability and natural products accumulation of medicinal plant *Sophora alopecuroides* L. based on MaxEnt model

Wenwen Rong [1,†], Xiang Huang [1,†], Shanchao Hu [1], Xingxin Zhang [2], Ping Jiang [1], Panxin Niu [1], Jinjuan Su [1], Mei Wang [1,*], and Guangming Chu[1,*]

1 Agricultural College, Shihezi University, Shihezi, Xinjiang 832003, China

2 College of Grassland Science, Xinjiang Agricultural University, Urumqi, Xinjiang 830052, China

* Correspondence: shzu_wm@163.com; cgmshzu@163.com;

† These authors contributed equally to this work.

1 Supplementary Figures and Tables

1.1 Supplementary Figures

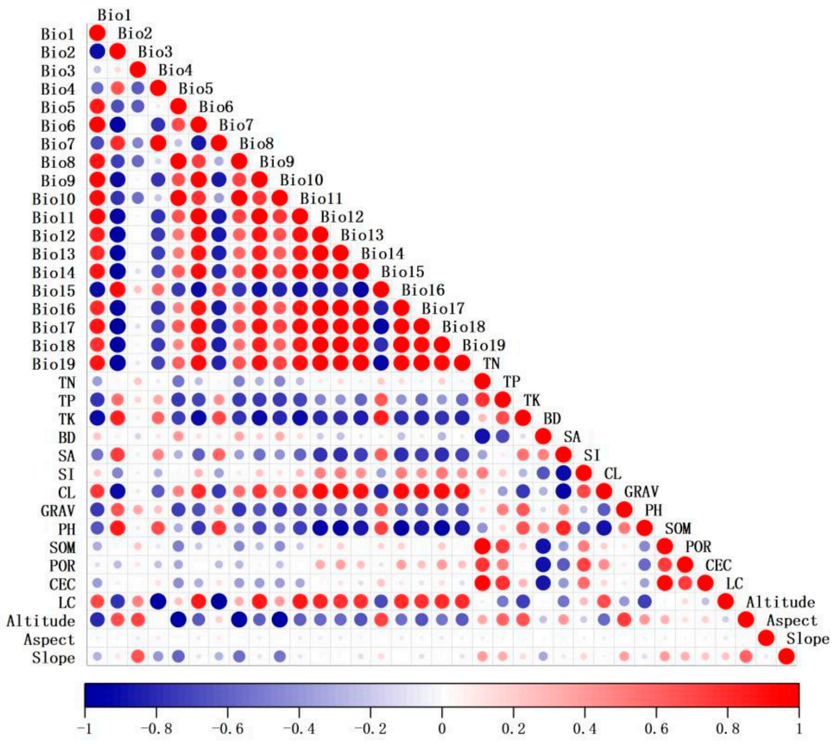

Supplementary Figure S1. Correlation analysis of 35 environment variables.

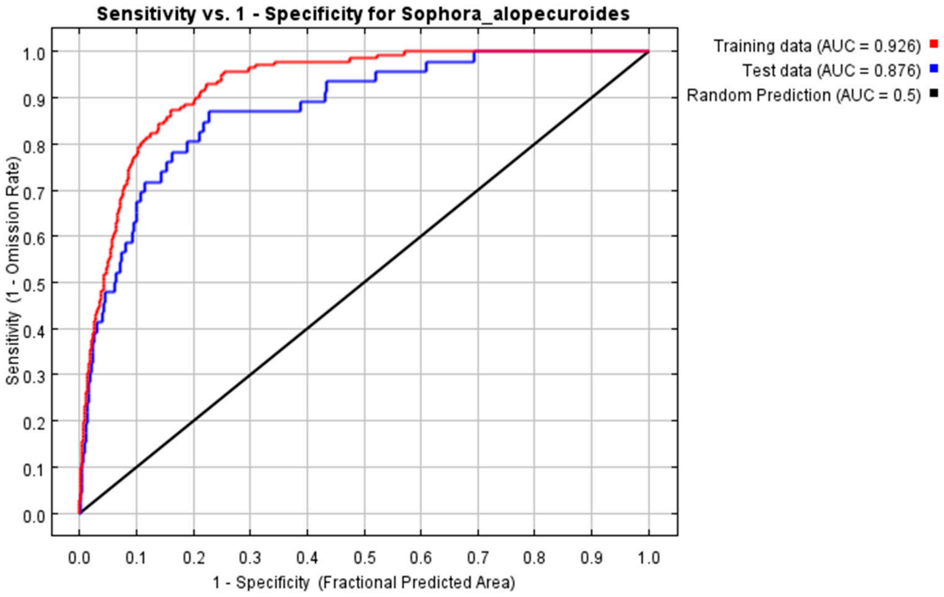

Supplementary Figure S2. The ROC curve of *S. alopecuroides* without human interference in the current period.

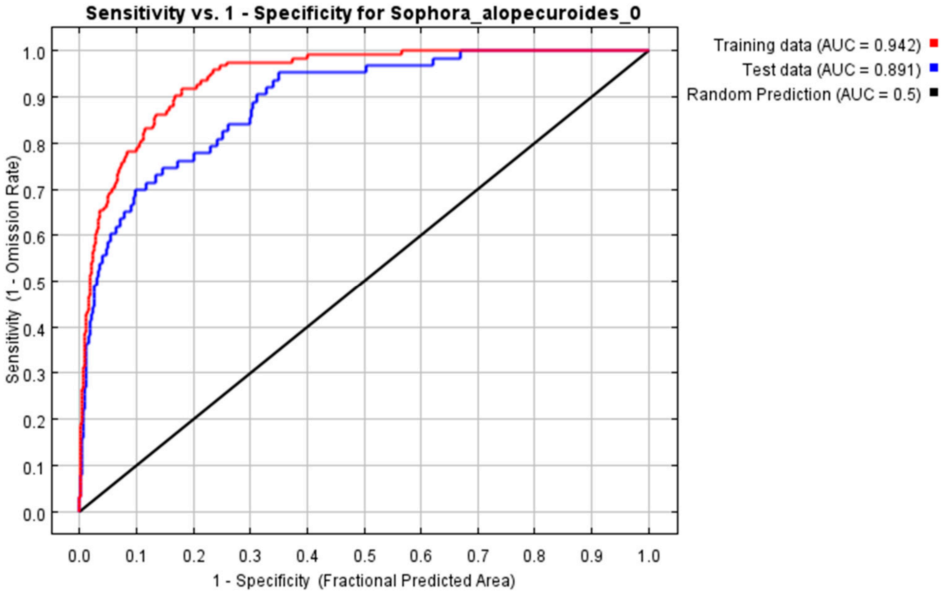

Supplementary Figure S3. The ROC curve of *S. alopecuroides* under human disturbance in the current period.

1.2 Supplementary Tables

Supplementary Table S1. 36 independent environmental variables.

Data type	Variable	Description	Unit
Climate variables	Bio1	Annual Mean Temperature	℃
	Bio2	Mean Diurnal Range	℃
	Bio3	Isothermality	%
	Bio4	Temperature Seasonality	℃
	Bio5	Max Temperature of Warmest Month	℃
	Bio6	Min Temperature of Coldest Month	℃
	Bio7	Temperature Annual Range	℃
	Bio8	Mean Temperature of Wettest Quarter	℃
	Bio9	Mean Temperature of Driest Quarter	℃
	Bio10	Mean Temperature of Warmest Quarter	℃
	Bio11	Mean Temperature of Coldest Quarter	℃
	Bio12	Annual Precipitation	mm
	Bio13	Precipitation of Wettest Month	mm
	Bio14	Precipitation of Driest Month	mm
	Bio15	Precipitation Seasonality	mm
	Bio16	Precipitation of Wettest Quarter	mm
	Bio17	Precipitation of Driest Quarter	mm
	Bio18	Precipitation of Warmest Quarter	mm
	Bio19	Precipitation of Coldest Quarter	mm
Soil variables	TN	Total Nitrogen	Mg/kg
	TP	Total Phosphorus	Mg/kg
	TK	Total Potassium	Mg/kg
	BD	Soil Bulk Density	g/cm
	SA	Sand Grains	wt%
	SI	Powder Grains	wt%
	CL	Sticky Particles	wt%
	GRAV	Gravel	wt%
	pH	Acidity and Basicity	—
	SOM	Organic Matter Content	%
	POR	Porosity	%
	CEC	Amount of Cation Exchange	mol/kg
	LC	Land use type	—

Terrain variables	Altitude	Altitude	m
	Aspect	Aspect	°
	Slope	Slope	°
Human activities	HF	Human Footprint	—

Supplementary Table S2. The percent contribution and permutation importance of 17 environment variables.

Variable	Percent contribution(%)	Permutation importance(%)
Bio1	16.8	14.3
Bio18	16.4	36.5
pH	11.5	3
LC	10	6.3
Altitude	8.7	7
Bio6	8.2	1.1
Bio15	8.1	3.8
Bio12	4.3	8.3
SI	3.8	2
GRAV	3.3	2.2
Bio4	3.3	3.8
Aspect	2.1	2
SOM	1.3	2.7
Slope	1.2	3.3
POR	0.6	0.5

Supplementary Table S3. Area of each suitable area in the future period.

Types of suitable areas	Area(km^2)			
	2021-2040	2041-2060	2061-2080	2081-2100
Total suitable area	3181705.52	3130391.15	3493885.83	3396696.26
Highly suitable area	471491.01	490742.85	554009.10	526169.08
Moderately suitable area	791686.94	735935.35	904549.93	880932.44
Lowly suitable area	1918527.56	1903712.96	2035326.79	1989594.74
